# Supplementary material for: Integration of mRNA and miRNA Analysis Reveals the Post-Transcriptional Regulation of Salt Stress Response in Hemerocallis fulva
Source: Int J Mol Sci. 2023 Apr 14;24(8):7290. doi: 10.3390/ijms24087290 (PMC10139057; doi:10.3390/ijms24087290)
Supplement: Supplementary file 1 [file ijms-24-07290-s001.zip › Supplementary figures.pdf]

Supplementary figures

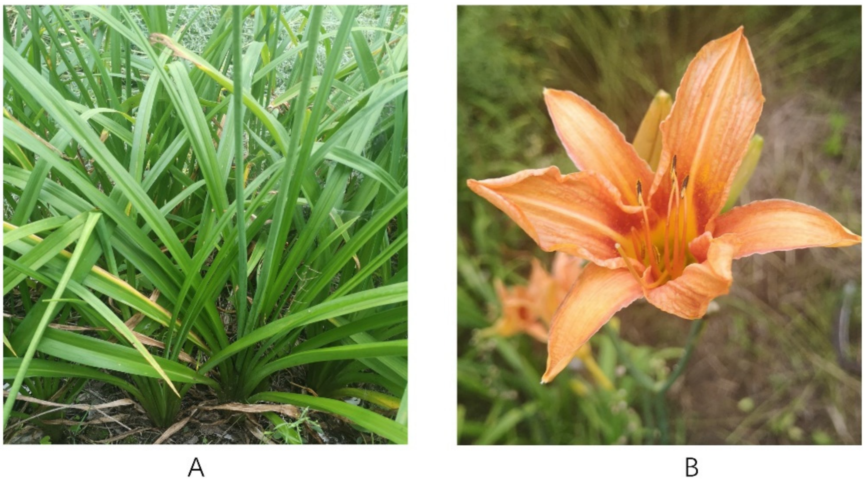

Figure S1 The cultivated variety 'Chengsebaoshi' of *H. fulva*. A, leaves of *H. fulva*; B, flower of *H. fulva*

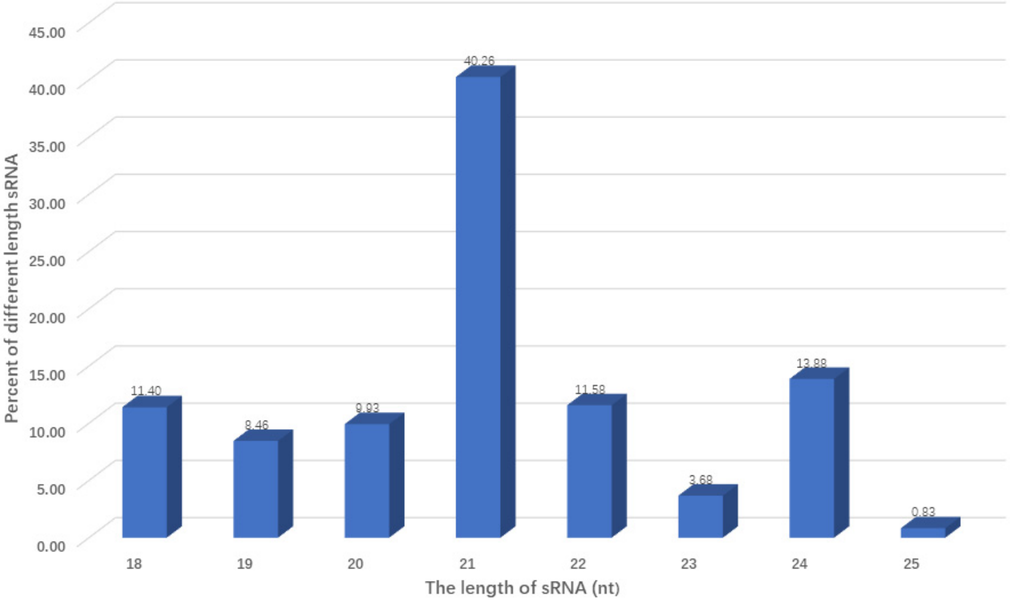

Figure S2 The distribution of different length sRNA in the sequencing libraries of *H.fulva*

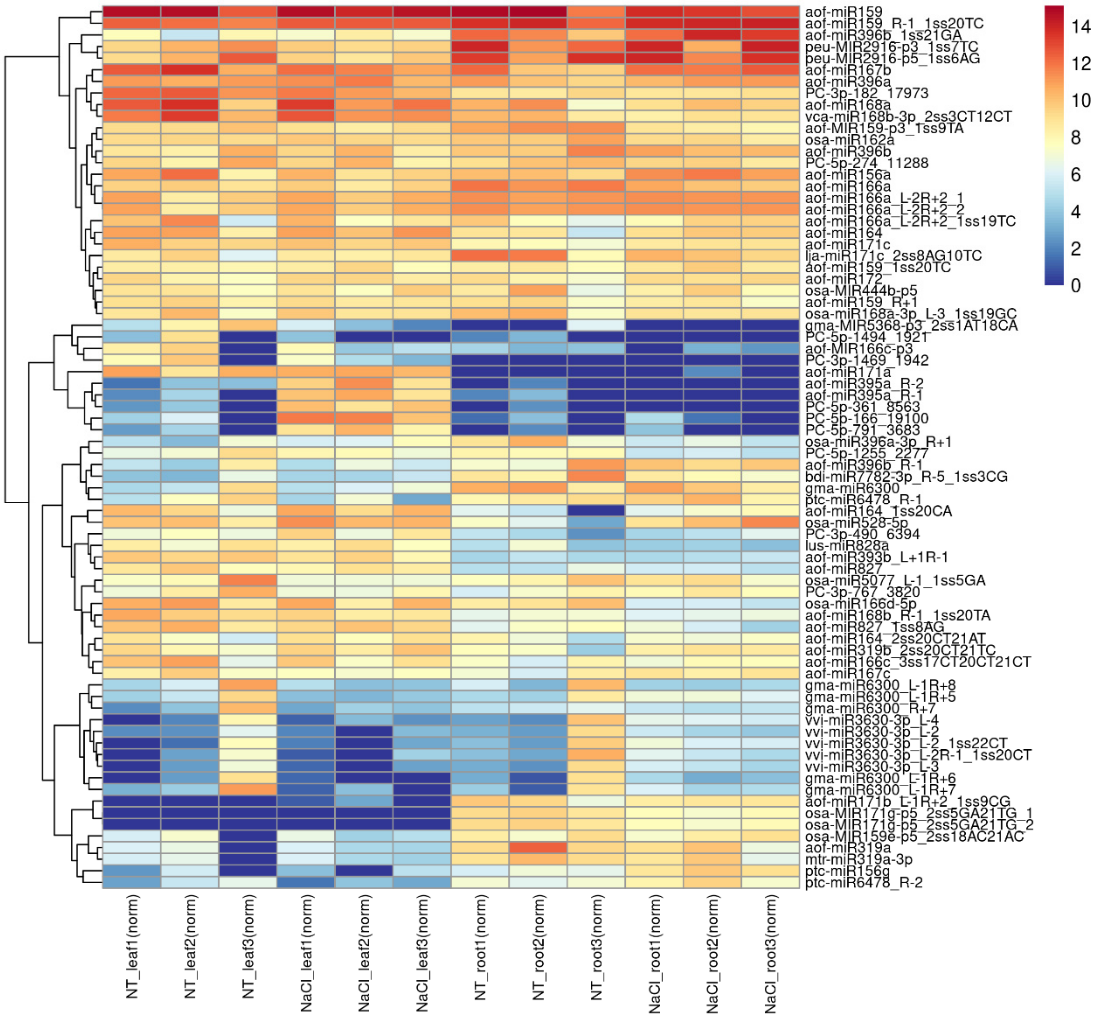

Figure S3 The heatmap analysis of transcription level of identified candidate miRNAs in leaf and root of *H. fulva* high throughput sequencing

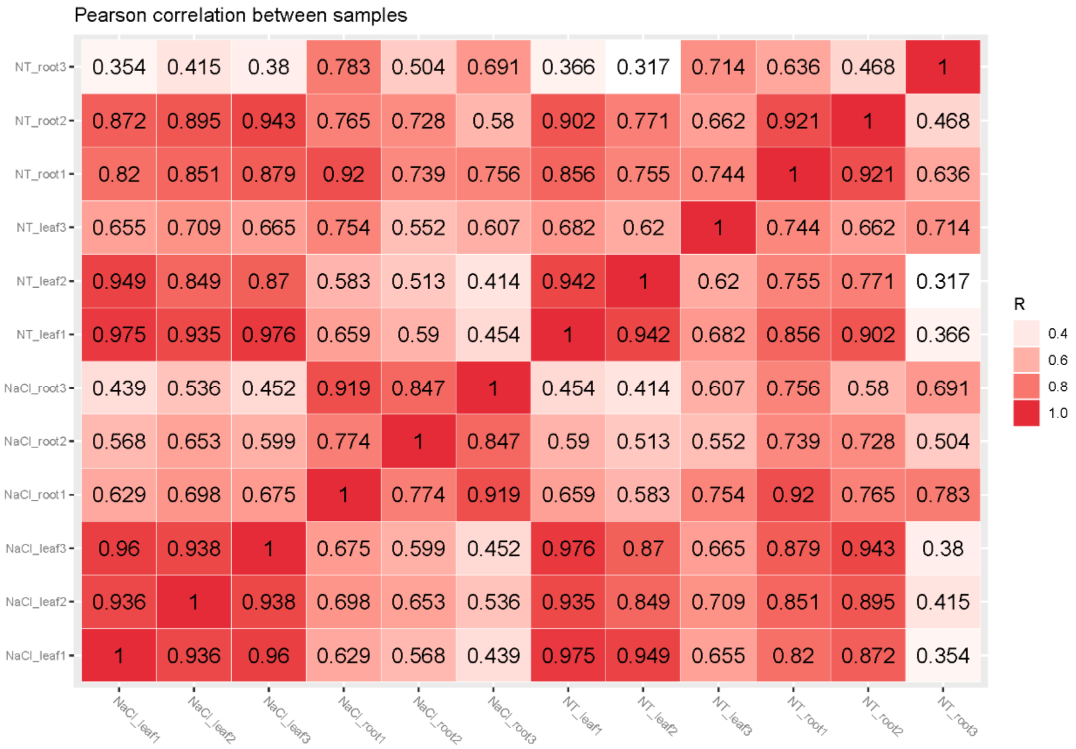

Figure S4 The Pearson correlation analysis based on the normalized expression levels between the samples of leaf and root with and without NaCl treatment in *H. fulva*

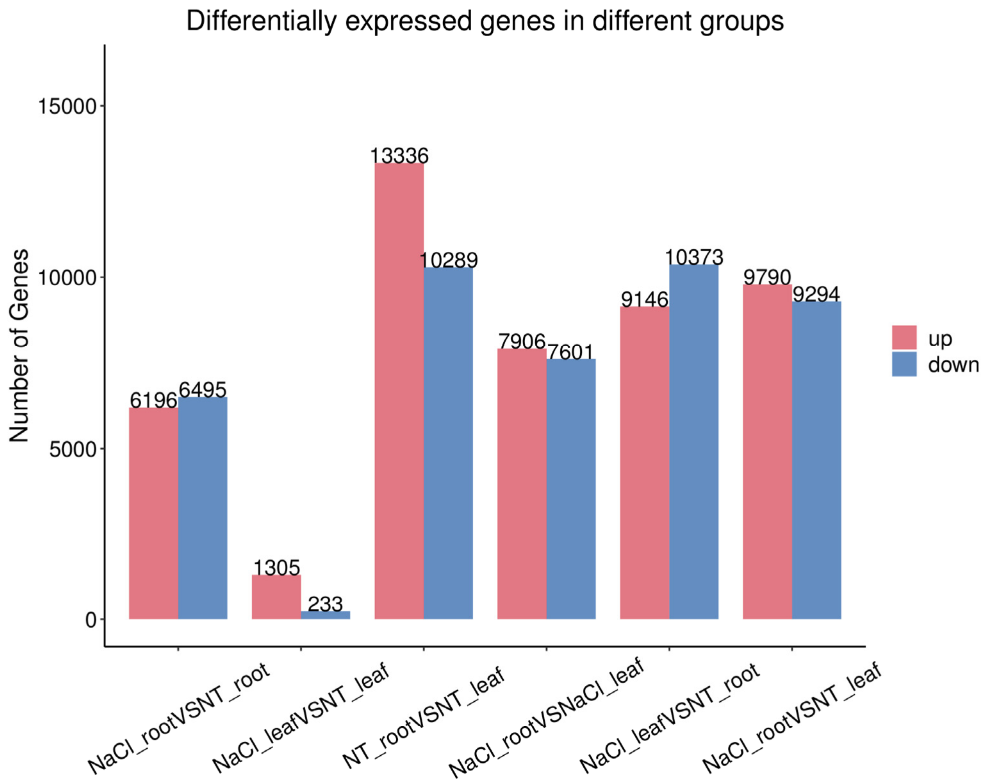

Figure S5 The bar plot showing the up- and down-differentially expressed genes in leaf and root under NaCl stress in *H. fulva*

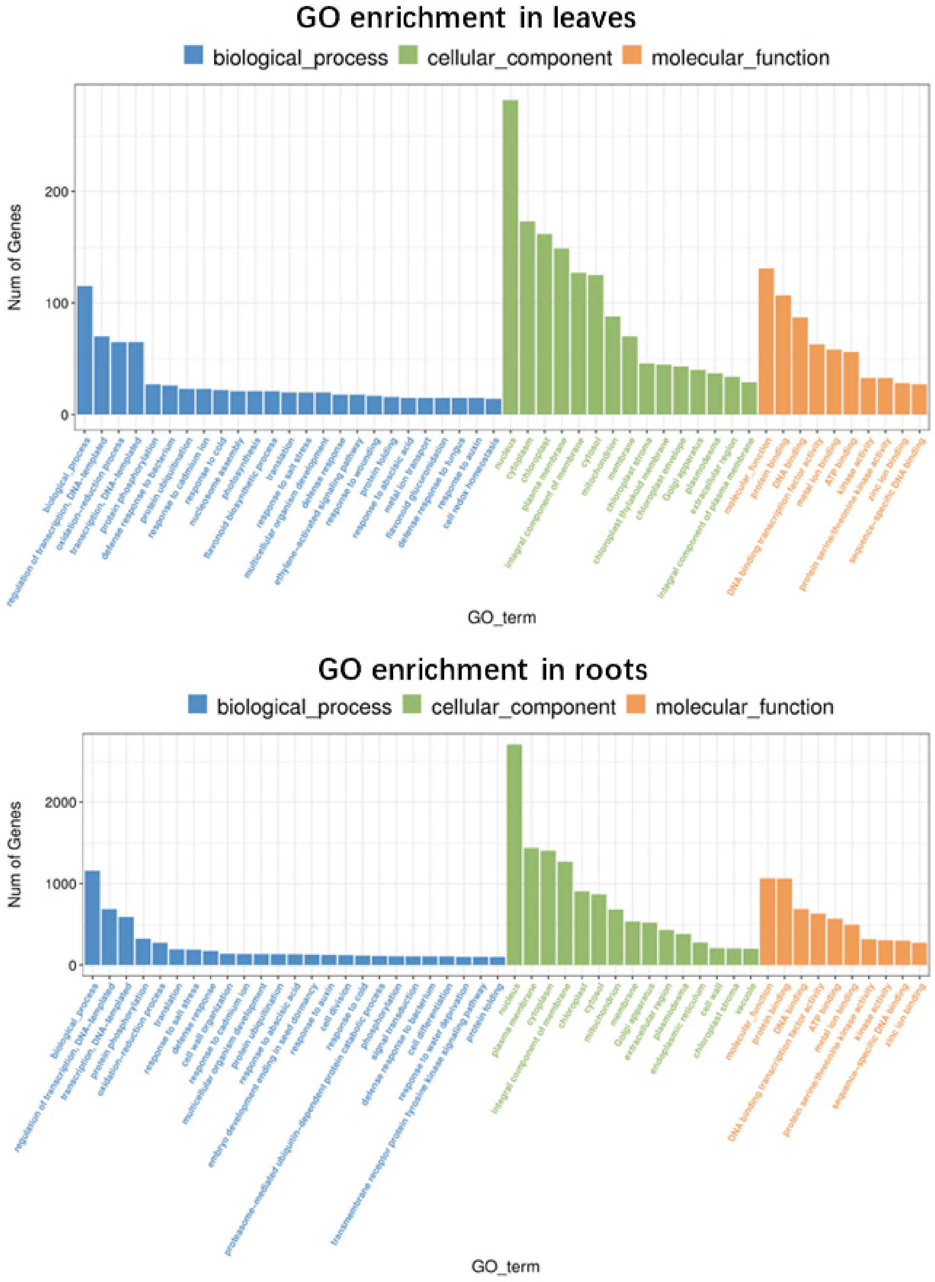

Figure S6 GO enrichment analysis of NaCl-stress responsive genes in leaf and root of *H. fulva*

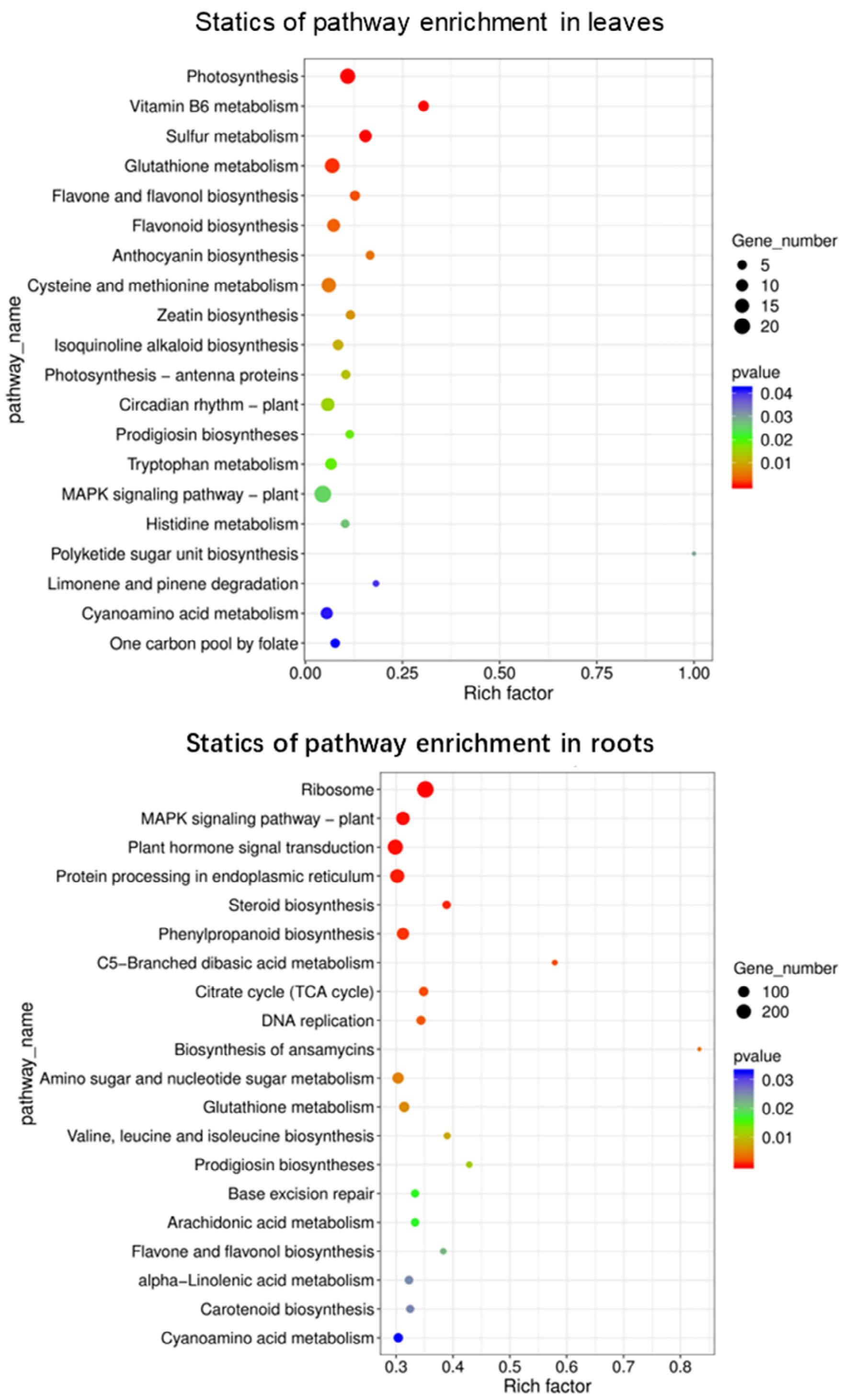

Figure S7 KEGG pathway analysis for the DE-mRNAs in leaf and root of *H. fulva*

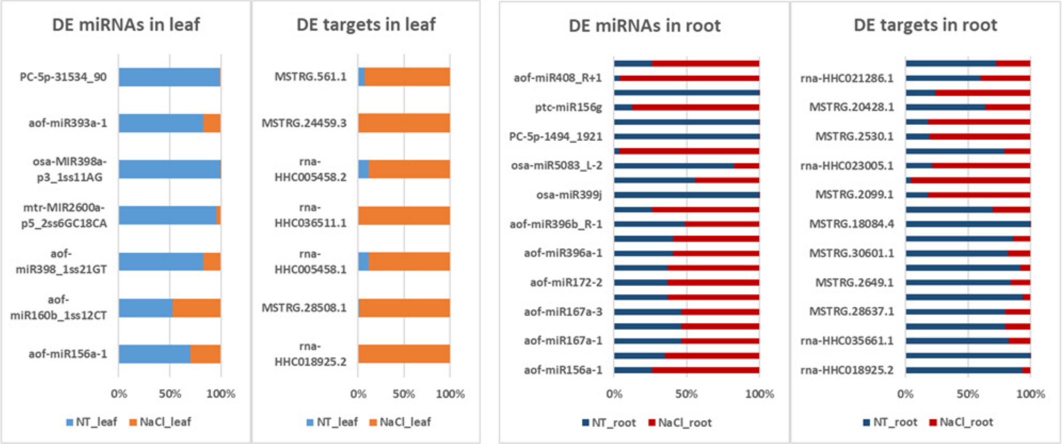

Figure S8 The opposite expression patterns of miRNA::targets pairs in leaf and root of *H. fulva*
