# Supplementary material for: Integration of mRNA and miRNA Analysis Reveals the Post-Transcriptional Regulation of Salt Stress Response in Hemerocallis fulva
Source: Int J Mol Sci. 2023 Apr 14;24(8):7290. doi: 10.3390/ijms24087290 (PMC10139057; doi:10.3390/ijms24087290)
Supplement: Supplementary file 1 [file ijms-24-07290-s001.zip › Table S1.pdf]

Table S1 Primers for real-time reverse transcription-PCR validated miRNAs and targets

Gene ID	Reverse transcription primer sequence	Forward primer sequence
HFmiR393a Stem-loop Primer	GTCGTATCCAGTGCAGGGTCCGAGGTATTCGCACTGGATACGACGAATCA	CGCCAGTCCAAAGGGATCGCAT
HFmiR396b Stem-loop Primer	GTCGTATCCAGTGCAGGGTCCGAGGTATTCGCACTGGATACGACAGTTCA	CGCCAGTTCCACAGCTTTCT
HFmiR160b Stem-loop Primer	GTCGTATCCAGTGCAGGGTCCGAGGTATTCGCACTGGATACGACTGGCAT	ACTCAATGCCTGGTTCCTTGT
HFmiR156a Stem-loop Primer	GTCGTATCCAGTGCAGGGTCCGAGGTATTCGCACTGGATACGACGTGCTC	CGCCAGTGACAGAAGAGAGT
RT-Universal Reverse Primer	CCAGTGCAGGGTCCGAGGTA	
HFACTIN	ATCACGGATGGCTGGAAGAGGAC	CTGAGCGGGAAGTTGTAAGGGAC
HFGRF4	AGGATTGGGTCTGGCTCTCG	GGATGAGGTGGATGGGTACG
HFTIR1	GGTTGCCCAAAGTTGACCAC	TGGTAAGAAGGCCGGAGAGA
HFARF18	CTTGGACTCTCAGCTCTGGC	TCATTTCCTCGGATCGGCTG
HFSPL12	AAGCTGCCGTAGGCGTTTAT	ATGAGTGATGCTGAAGCCCC
